# Understanding Spatio-Temporal Variability in the Reproduction Ratio of the Bluetongue (BTV-1) Epidemic in Southern Spain (Andalusia) in 2007 Using Epidemic Trees

**DOI:** 10.1371/journal.pone.0151151

**Published:** 2016-03-10

**Authors:** S. Napp, A. Allepuz, B. V. Purse, J. Casal, I. García-Bocanegra, L. E. Burgin, K. R. Searle

**Affiliations:** 1 Centre de Recerca en Sanitat Animal (CReSA)—Institut de Recerca i Tecnologia Agroalimentàries (IRTA), Campus UAB, 08193 Bellaterra, Barcelona, Spain; 2 Departament de Sanitat i Anatomia Animals, Universitat Autònoma de Barcelona (UAB), 08193 Bellaterra, Barcelona, Spain; 3 Centre for Ecology and Hydrology, MacLean Bldg, Benson Lane, Crowmarsh Gifford, Wallingford, Oxfordshire, OX10 8BB, United Kingdom; 4 Departamento de Sanidad Animal, Facultad de Veterinaria, Universidad de Córdoba (UCO), Campus Universitario de Rabanales, 14071 Córdoba, Spain; 5 Met Office, FitzRoy Road, Exeter, Devon EX1 3PB United Kingdom; 6 Centre for Ecology and Hydrology, Bush Estate, Penicuik, Midlothian, EH26 0QB, United Kingdom; Institut Pasteur, FRANCE

## Abstract

Andalusia (Southern Spain) is considered one of the main routes of introduction of bluetongue virus (BTV) into Europe, evidenced by a devastating epidemic caused by BTV-1 in 2007. Understanding the pattern and the drivers of BTV-1 spread in Andalusia is critical for effective detection and control of future epidemics. A long-standing metric for quantifying the behaviour of infectious diseases is the case-reproduction ratio (*R*_*t*_), defined as the average number of secondary cases arising from a single infected case at time *t* (for *t*>0). Here we apply a method using epidemic trees to estimate the between-herd case reproduction ratio directly from epidemic data allowing the spatial and temporal variability in transmission to be described. We then relate this variability to predictors describing the hosts, vectors and the environment to better understand why the epidemic spread more quickly in some regions or periods. The *R*_*t*_ value for the BTV-1 epidemic in Andalusia peaked in July at 4.6, at the start of the epidemic, then decreased to 2.2 by August, dropped below 1 by September (0.8), and by October it had decreased to 0.02. BTV spread was the consequence of both local transmission within established disease foci and BTV expansion to distant new areas (i.e. new foci), which resulted in a high variability in BTV transmission, not only among different areas, but particularly through time, which suggests that general control measures applied at broad spatial scales are unlikely to be effective. This high variability through time was probably due to the impact of temperature on BTV transmission, as evidenced by a reduction in the value of *R*_*t*_ by 0.0041 for every unit increase (day) in the extrinsic incubation period (*EIP*), which is itself directly dependent on temperature. Moreover, within the range of values at which BTV-1 transmission occurred in Andalusia (20.6°C to 29.5°C) there was a positive correlation between temperature and *R*_*t*_ values, although the relationship was not linear, probably as a result of the complex relationship between temperature and the different parameters affecting BTV transmission. *R*_*t*_ values for BTV-1 in Andalusia fell below the threshold of 1 when temperatures dropped below 21°C, a much higher threshold than that reported in other BTV outbreaks, such as the BTV-8 epidemic in Northern Europe. This divergence may be explained by differences in the adaptation to temperature of the main vectors of the BTV-1 epidemic in Andalusia (*Culicoides imicola*) compared those of the BTV-8 epidemic in Northern Europe (*Culicoides obsoletus*). Importantly, we found that BTV transmission (*R*_*t*_ value) increased significantly in areas with higher densities of sheep. Our analysis also established that control of BTV-1 in Andalusia was complicated by the simultaneous establishment of several distant foci at the start of the epidemic, which may have been caused by several independent introductions of infected vectors from the North of Africa. We discuss the implications of these findings for BTV surveillance and control in this region of Europe.

## Introduction

The basic reproduction ratio (*R*_*0*_) is the most widely used parameter in epidemic theory and is a key tool for understanding the behaviour of infectious diseases [1]. It is defined as the average number of secondary cases produced when a single infected individual is introduced into a fully susceptible population [2]. As the epidemic progresses, as a result of the depletion of susceptible animals or the application of control measures, the basic reproduction ratio changes to the case reproduction ratio (*R*_*t*_), i.e. the average number of secondary cases arising from a single infected case at time *t* (for *t*>0). Knowledge of *R*_*t*_ is very relevant for the control of the epidemic [3]. If *R*_*t*_<1, each case will, on average, produce less than one secondary case, and the epidemic will tend to die out, even if no further measures are applied. However, if *R*_*t*_>1, each case will, on average, produce more than one secondary case, and extra measures will be needed to control the epidemic. The proportion of the population that would need to be protected to achieve the eradication of the disease (*p*) can be estimated as: *p>*1-(1/*R*_*t*_) [2]. Therefore, the higher the value of *R*_*t*_, the more difficult it will be to control the epidemic. Both *R*_*0*_ and *R*_*t*_ are usually derived from explicit deterministic susceptible-infectious-recovered (SIR) models, and estimated by fitting a equations to epidemic (case or seroprevalence) data [4,5]. However, there are two problems associated with this approach. First, many assumptions need to be made, for example about the size of the susceptible population, which in the context of an epidemic is continuously expanding. Second, *R*_*0*_ and *R*_*t*_ are estimated as mean values, without consideration of how these parameters vary over both space and time, thereby excluding spatio-temporal information of considerable epidemiological importance [6]. To overcome these difficulties, Haydon and collaborators [4] developed a method based on the construction and analysis of epidemic trees, which has the advantage that the case-reproduction ratio can be estimated directly from epidemic data [7].

Bluetongue is a viral disease caused by Bluetongue virus (BTV), which belongs to the genus *Orbivirus* within the family *Reoviridae*. Traditionally, 24 different serotypes have been classified, but in recent years, two new serotypes BTV-25 and BTV-26 have been identified [8], reflecting the dynamic nature of this disease. Although bluetongue affects all ruminant species, severe disease is mainly restricted to certain breeds of sheep [9]. BTV is transmitted between hosts almost exclusively by the bites of certain species of *Culicoides* biting midges (Diptera: Ceratopogonidae).

In 2007, Andalusia, the southernmost region of Spain, was affected by a devastating epidemic caused by bluetongue virus serotype 1 (BTV-1) resulting in more than four thousand infected farms [10]. Besides BTV-1, Andalusia has been affected by several other BTV serotypes: BTV-10 was introduced in 1956, BTV-4 in 2004 and BTV-8 in 2008 [11]. Except in the case of BTV-8, the other serotypes were likely to have been introduced through wind transportation of infected *Culicoides* from the North of Africa [11]. Introduction of bluetongue from Northern Africa into Southern Spain is considered to be one of the main routes of introduction of new serotypes of bluetongue into Europe [12]. Therefore, understanding the pattern of BTV spread in Andalusia is critical for facilitating effective surveillance and control of future epidemics.

The objective of this study was to calculate the between-herd case-reproduction ratio (*R*_*t*_) of the BTV-1 epidemic in Andalusia in 2007 using epidemic trees, and to describe the main factors that determined spatial and temporal variation in its value.

## Materials and Methods

### 1. Data collection

Andalusia is a region located in the south of Spain and has an area of 87597 km^2^. The ruminant population comprises approximately 2.6 million sheep, 1.2 million goats and 0.6 million cattle, divided into 13346 sheep flocks, 10789 goat flocks, 8745 cattle herds and 4339 mixed farms.

Epidemiological data on the 2007 BTV outbreak was collected by the Veterinary Services of the Autonomous Government of Andalusia. A case was defined as a farm where BTV-1 was confirmed by the national reference laboratory as positive in at least one animal (sheep, goat or cattle) by means of RT-PCR analysis according to the OIE guidelines. As detection relied on previous clinical detection, only seven cattle herds affected by BTV-1 were detected in the epidemic [10], and therefore they were excluded from our analysis. A total of 4421 infected farms, 3699 in small ruminant flocks and 722 in mixed farms (sheep, goat and/or cattle in the same farm) were included in the analysis.

### 2. Determination of the date of infection and the date of onset of infectiousness of the affected farms (Step 1 in S1 Fig & S2 Fig)

For the first 974 farms affected during the epidemic, questionnaires were conducted so that the date when clinical symptoms were first observed, the date of BTV notification to the authorities and the date of BTV confirmation at the farms were recorded. That information allowed the average delay between the detection of clinical symptoms at the farm and BTV confirmation to be calculated (S2 Fig). Then, for the remaining farms, the approximate date of development of clinical symptoms could be estimated based on its date of BTV confirmation.

In sheep, the average incubation period is between 6 and 8 days [13], so a conservative delay of twice that period (14 days) was considered between the infection of the farm and the detection of the clinical signs by the farmer. By subtracting these 14 days from the estimated date of development of clinical symptoms, the approximate date of infection of each of the 4421 farms was estimated.

From the date of infection of the farm, the date of onset of infectiousness on that farm may be calculated, taking into account [14]:

1. The time from infection to viraemia (*TITV*) in the hosts, between 1 and 6 days [15].
2. The extrinsic incubation period (*EIP*) in the vectors.

The *EIP* is defined as the time between the infection of the vector and when it first becomes capable of transmitting the virus, and was calculated as the reciprocal of the virogenesis rate. The virogenesis rate (*v*) depends on the temperature (*T*), and may be estimated as [16]:

v=v(T)

$$\left\{ \begin{array}{c} 0\;T<T_{min}\\ \alpha (T-T_{min})\;T>T_{min} \end{array}\right.$$

where *T*_*min*_ is the threshold temperature for virus replication, which for BTV-1 in *C*. *imicola* (the main vector of BTV in Andalusia) was estimated as 12.7°C; and *α* represents the mean rate of replication above the threshold temperature, which for BTV-1 in *C*. *imicola* was estimated as 0.016 per degree-day.

For the estimation of the *EIP*, the mean daily temperatures at the point location of each farm, for 50 days after the date of infection of the farm were obtained. Temperature data was taken from the UK Met Office’s Numerical Weather Prediction (NWP) model, the Unified Model [17]. For the time period of this study data is available from the European domain of the model at a horizontal resolution of 12km. Hourly values of temperature at the surface were extracted to the point location of each farm and from these daily mean temperatures were calculated. The virogenesis rate on a given day (i.e. for a given mean temperature) represented the proportion of the *EIP* completed that day [14]. The duration of the *EIP* on that farm was given by the number of days required for the summation of these proportions to reach one (i.e. for the *EIP* to be completed). For each infected farm, the day a farm got infected, plus the *TITV*, plus the number of days needed to complete the *EIP*, determines the day from which a given farm became infectious. Because the animals within a farm are infected (and then become infectious) progressively over time, once a farm became infectious, it was assumed to remain infectious until November (when the last infections of the epidemic occurred).

### 3. Development of the epidemic trees (Step 2 in S1 Fig)

For each of the infected farms (daughter farms), the source of its infection (parent farm) was selected using an algorithm. The majority of the results presented in this paper were based on an algorithm in which the parent farm was assigned to the closest infectious farm (algorithm 1), as in Haydon and collaborators [4], however we also considered two other types of algorithms:

1. One in which all infectious farms within a given distance (5 km) of the daughter farm were assumed to have the same probability of having been the parent farm (algorithm 2). The choice of the distance was based on Sedda and collaborators [18], who found that in the BTV-8 epidemic in Northern Europe, the majority of infections occurred over distances of 5 km or less.
2. Another in which all infectious farms within 5 km of the daughter farm were considered as potential parent farms, but their probabilities were inversely proportional to their distance (algorithm 3).

One advantage of the method developed by Haydon and collaborators [4] is that it allows the case-reproduction ratios to be estimated for different areas and periods. Similarly, the BTV-1 epidemic in Andalusia can be viewed as sets of clades within which transmission was local. The difficulty lies in differentiating local transmission from a new focus (i.e. how far from the closest infectious farm a new infection can still be considered as local transmission, versus when it should be considered as the onset of a new focus). In order to define this distance, we estimated for each affected farm, the distance to the nearest infectious farm, and used the 99^th^ percentile of these distances, (52.1 kilometers) to set as the spatial limit to differentiate local transmission from a new focus.

### 4. Estimation of the case-reproduction ratio (*R*_*t*_)

Once the parent farm (i.e. the farm responsible for the infection) had been assigned to each infected farm by means of one of the algorithms, we calculated the number of farms infected by each of the 4421 farms affected throughout the epidemic: *N*_*i*_ (Step 3 in S1 Fig). Then, the case-reproduction ratio (*R*_*t*_) for a given focus (*x*) and time period (*t*) was calculated as the average value of *N*_*i*_ of the farms infected in focus *x* during the period *t* (Step 4 in S1 Fig).

The algorithms were programmed in R software [19]. Maps were generated using QGIS [20].

### 5. Assessment of the influence of different factors on the value of *R*_*t*_

The relative influence of different factors on the value of *R*_*t*_ was assessed by means of a statistical model. For each time step, a buffer of 5 km around the farms that belonged to each focus was created. These buffers were used to extract the mean values of environmental variables from different raster layers.

Variables assessed included (S1 Table):

1. Variables related to the host species: density of domestic hosts, i.e. cattle, sheep and goats per km^2^, and in relation to wild hosts: red deer habitat suitability [21] and roe deer habitat suitability [22]. Two new variables, total domestic ruminants (cattle, sheep and goats) and total wild ruminants (red deer and roe deer) were also created. Data on the population of domestic ruminants in a raster format was provided by the Autonomous Government of Andalusia.
2. Variables related to the vector species: the median and the 95% upper limit of the credible interval of predicted seasonal maximum abundance of *C*. *imicola*, *C*. *obsoletus* and *C*. *pulicaris* (the three main bluetongue vector species in Andalusia) within the buffer area. Two new variables, median and 95% upper limit of the CI of *Culicoides* captures (sum of the 3 species) were also created.

Models for the maximum abundance of *Culicoides* vector species were developed from national surveillance datasets of weekly OVI light trap catches at approximately 300 sites across the UK and Spain covering a five year period (2005–2010) [23]. Briefly, at 404 site by year combinations, five *Culicoides* vector species or species complexes were identified morphologically and counted (*C*. *imicola*, *C*. *newsteadi*, *C*. *impunctatus*, *C*. *obsoletus* complex, and *C*. *pulicaris* complex). For each site and year the maximum size of weekly female catch per species per year was determined. Only sites that had intense seasonal coverage of trapping, defined as no more than 2 weeks missing per year, were used in the analysis.

The analysis involved fitting Poisson Generalised Linear Mixed Models (GLMM) using Integrated Nested Laplacian Approximation (INLA) to identify the best-fitting models using a range of environmental covariates, and random effects for site and year. All possible combinations of explanatory variables were tested over 20 splits in the dataset into testing (75%) and training (25%) subsamples. An out of sample predictive r^2^ was calculated for each of the 20 splits in the data for each combination of explanatory variables for each species or species complex. These 20 out of sample predictive r^2^ values per combination were then averaged to give a final out of sample statistic for each possible combination of explanatory variables. Finally, all possible combinations of variables were ranked according to their out of sample predictive ability and the top 20 best models were selected for each species to make predictions at new sites across western Europe [23].

Predictions for the maximum annual abundance from each of the top 20 models (based on out of sample prediction performance) were averaged for each species, and weighted by their relative support in the data. Predictions were only made for pixels that were environmentally similar to the training data set, defined as those with a Malhalanobis Distance (MD) falling within the 0–80% cumulative distribution of MDs from the complete training data set (404 site by year combinations from the UK and Spain). Uncertainty in the estimation of model parameters was properly accounted for in the final predictions by using multiple samples from the posterior distribution of each model parameter and averaging across multiple predictions to generate a final averaged prediction. All predictions included a 95% credible interval [23].

3 Other variables

Median *EIP*s in the affected farms, were also obtained. *EIP* represents a measure of the favourability of temperatures for BTV transmission.

Intuitively, *R*_*t*_ within a specific focus is likely to decrease as the time since the initiation of that focus progresses. Therefore, a variable to account for the time since the start of each focus was created, defined as the days elapsed since the earliest date of infection amongst the farms in the focus.

As the epidemic expands and progresses, some foci grow and end up overlapping others. In these overlapping areas *R*_*t*_ values are likely to be lower as the farms that get the infection are shared by different foci. Therefore, a variable to account for the proportion of a given focus area at a given time period, overlapped by other foci was also created.

Land cover, elevation and slope were also included as variables to be assessed. Elevation was obtained from SRTM data [24] at 85.7m. Using the terrain function of the raster package in R [19], slope and aspect were calculated from the elevation layer. Furthermore, a terrain ruggedness index (TRI), defined as the mean of the absolute differences between the value of a cell and the value of its eight surrounding cells, was calculated for both slope and elevation with higher values of these indices indicating a wider variability in slope or elevation in the surrounding area, which we hypothesised might make it more difficult for infected midges to move around the landscape. Land cover data were obtained from Corine Land Cover 2006 at a 100m resolution and used to create an index of the suitability of landcover for movement of infected midges around the landscape. Landcover categories that were wholly unsuitable for farms or midges were assigned a high cost of movement value (2) while agricultural or grassland/shrubland categories with a high percentage of farms were assigned the lowest cost of zero. The intermediate cost of movement category, assigned a value of one, contained agricultural or grassland/shrubland categories which had a lower percentage of farms.

S2 Table includes the cost category assigned to each individual Corine Land Cover class. Having reclassified the Corine Land Cover Map according to these cost categories, the cost of movement were averaged at a 1km grid cell level. As stated above, these landscape factors were extracted for a 5km buffer zone around the farms in each focus at each time step.

#### Statistical analysis

Due to the hierarchical nature of the data, a mixed-effects model was used within the nlme library [25] implemented in R [19]. Because the different foci were observed through time, a nested random factor where focus was nested within time was included in the model. The Spearman Rank Correlation Coefficient was used to assess the correlation between the different environmental predictors included in the analysis. In those cases where the correlation was higher than 0.9 the variable with the higher biological significance was retained. The model was run with all the predictors, and full subset model selection was performed using REML comparing all possible subsets of environmental predictors. Models were compared using AICc [26].

### 6. Assessment of the effect of temperature on the value of *R*_*t*_

The mean temperatures at the farms infected by BTV was calculated for each week since the start of the epidemic, and the relationship between those temperatures and the weekly *R*_*t*_ values for the whole epidemic (i.e., for the whole of Andalusia), was evaluated using the Pearson correlation coefficient.

## Results

First we evaluated how the case-reproduction ratio (*R*_*t*_) of the BTV-1 epidemic for the whole of Andalusia varied through the epidemic. Considering the natural months between July and November, and using the algorithm for the closest farm (algorithm 1), the *R*_*t*_ value reached its maximum value (4.6) in July, at the start of the epidemic, then decreased to 2.2 by August, and dropped below 1 by September (0.8). By October it had decreased to 0.02 and remained close to 0 in November. There were no significant differences in *R*_*t*_ values when algorithm 2 (equally proportional within a distance) was applied, except that values for July and August were a bit lower (3.3 and 1.9, respectively) and for September and October values were a bit higher (0.9 and 0.2, respectively). Similarly, *R*_*t*_ values when algorithm 3 (proportional to distance) was applied were analogous to those obtained using algorithm 1, but values for July were a bit lower (3.3) and for August were a bit higher (2.4). In contrast to the monthly values of *R*_*t*_, the number of infected farms reported per month went from 113 in July, to 1010 in August and reached a maximum in September (2089 infected farms). In October, despite the low value of *R*_*t*_, there was a significant number of infected farms reported (1192), which rapidly decreased to only 15 farms infected in November.

**Fig 1:**
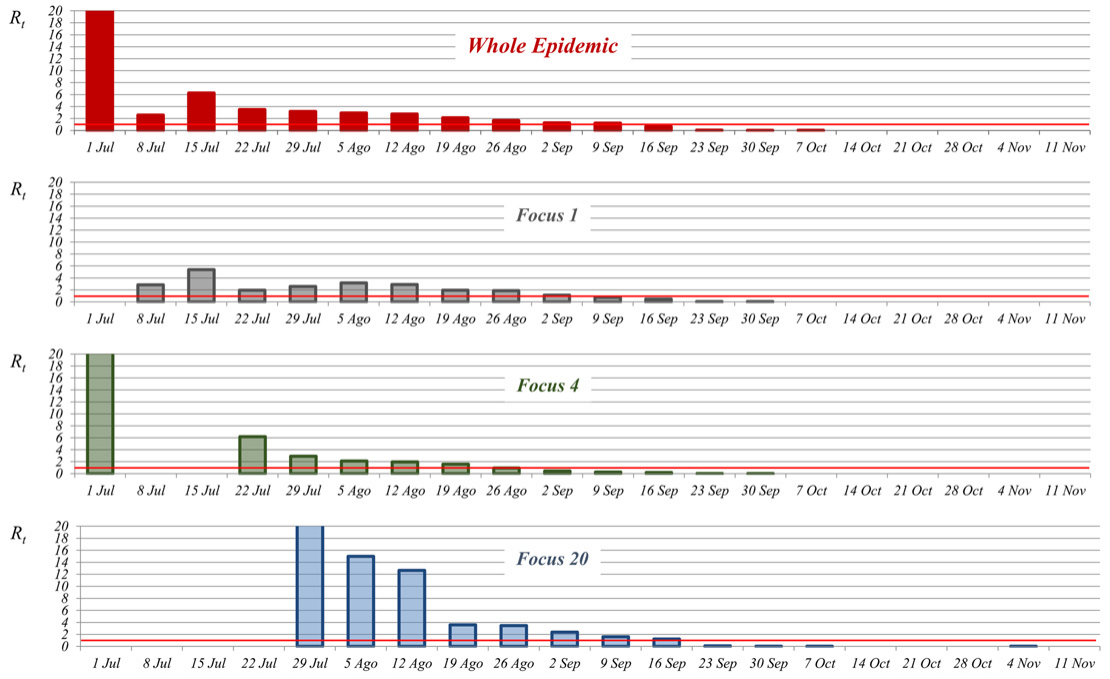
Temporal pattern of the weekly *R*_*t*_ values for the whole BTV-1 epidemic in Andalusia and for 3 of the main foci (focus 1, 4, 20) between week 1 (1^st^ of July) and week 20 (11^th^ of November).

If we use a smaller time step (i.e. one week) to evaluate the variation of the case-reproduction ratio for the whole of Andalusia, we observe a much higher heterogeneity in the values of *R*_*t*_, particularly at the start of the epidemic. Fig 1 shows how the first week of the epidemic *R*_*t*_ reached a very high value (20), decreased to 2.6 by week 2, increased to 6.3 in the third week (mid-July), and then there was a slow progressive decrease, so that it was not until week 12 (mid-September) that *R*_*t*_ fell below 1.

**Fig 2:**
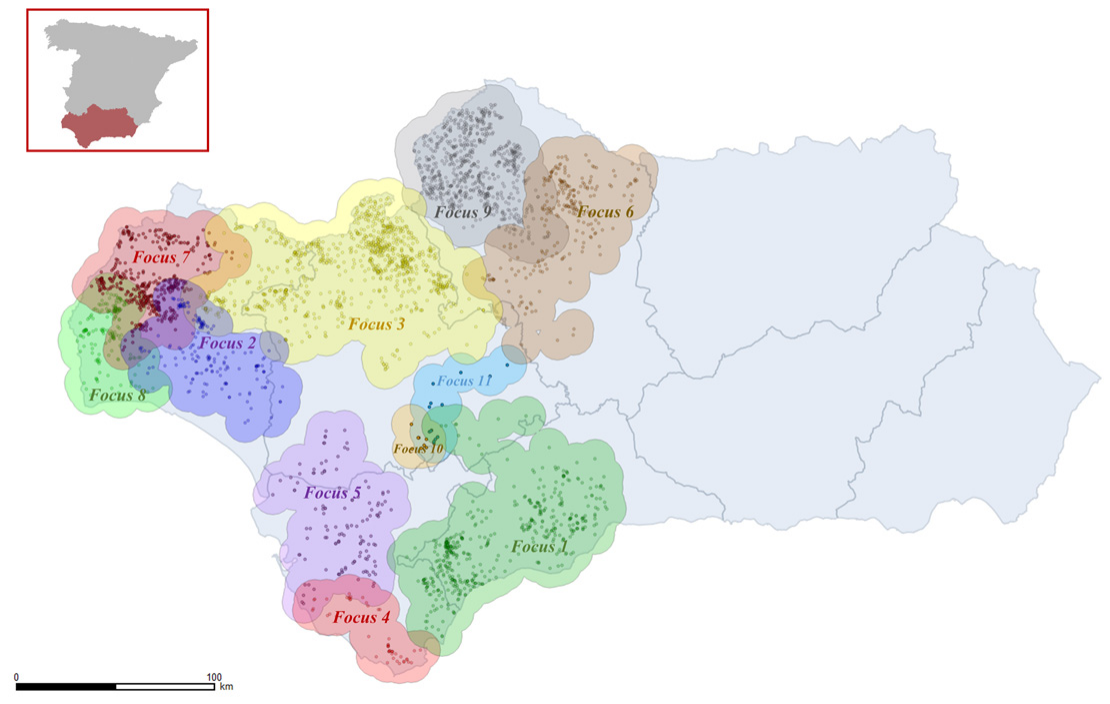
Spatial distribution of the farms affected plus buffer areas of the first 11 foci identified during the BTV-1 epidemic in Andalusia in 2007.

Looking at this smaller time-step (*R*_*t*_ values per week) it is easier to appreciate that the temporal pattern of the for the whole BTV-1 epidemic in Andalusia is the result of both the intensity of local BTV transmission within established foci and BTV expansion to new areas (i.e. new foci). If we look at the weekly estimations of *R*_*t*_ for the whole of Andalusia, the high value obtained in week 1 is likely to be the result of the *R*_*t*_ value in focus 4 (where the epidemic starts), while estimations for weeks 2 and 3 are probably linked to introduction and spread within focus 1, and the maintenance of high *R*_*t*_ values between week 4 (22^nd^ of July) and week 7 (12^th^ of August) are highly dependent on the introduction and spread within focus 20 (the largest focus in the epidemic) (Fig 1 and Fig 2).

**Fig 3:**
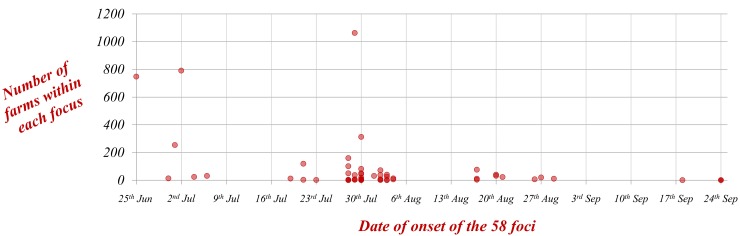
Number of infected farms within each of the 58 disease foci depending on the date of onset of the foci.

While in some of the foci (e.g. focus 4 in Fig 1), there was a delay of 1 or 2 weeks between the primary infection (as a result of BTV introduction) and the first secondary cases arising as a result of local spread, in other cases (e.g. focus 1 and 20 in Fig 1), there was no such a delay. In general, the foci initiated earlier in the epidemic tended to be larger (i.e. more farms infected) (Fig 3), and of longer duration than the foci initiated later. The results show that there was a great variation in the effectiveness of BTV spread among the 58 foci. In some foci, BTV-1 seemed to fail to establish a continuous transmission within the new area, and none or a few secondary cases were produced (e.g. foci 10 and 11 in Fig 2). In fact, of the 58 foci, 21 had 4 infected farms or less. However, in some other cases, BTV easily spread in the new area, and transmission was maintained for many weeks with hundreds of secondary cases, for example focus 4 (791 cases in 18 weeks) and focus 1 (748 cases in 18 weeks) (Fig 2). In some cases as a consequence of BTV local spread, foci ended up overlapping with each other (for example foci 2, 3, 7 and 8 in Fig 2).

**Fig 4:**
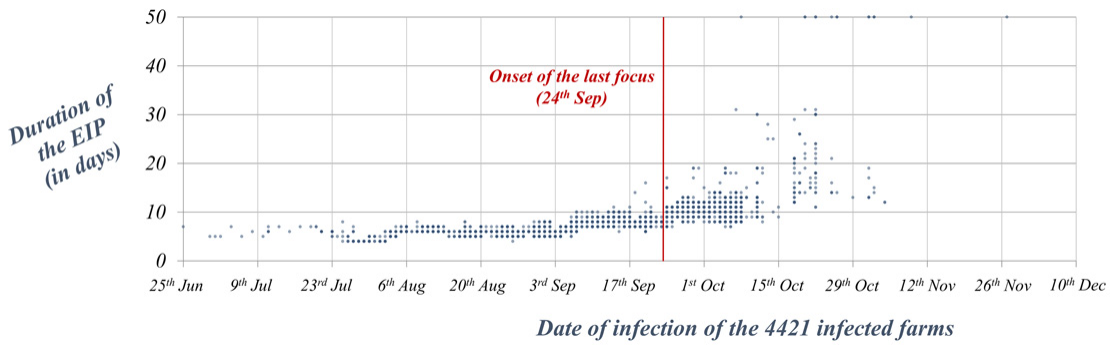
Duration of the *EIP* of the 4421 farms infected throughout the BTV-1 epidemic in 2007 in Andalusia depending on the date of infection. For representation purposes, farms where the *EIP* could not be completed were arbitrarily given a value of 50 days.

Between weeks 3 and 9 (end of August), the slow progressive decrease of *R*_*t*_ values, coincided with an even slower progressive increase of the time needed for the completion of the *EIP* in the affected farms (Fig 4). Between weeks 10 and 16 (mid-October), increase of *EIP*s becomes steeper, going from a mean of 5.7 days between weeks 3 and 9 to mean of 9.3 days between weeks 10 and 16, which results in a rapid decrease of *R*_*t*_ values, that after week 13 are close to 0 (Fig 2 and Fig 4). After week 16 (mid-October), the *EIP*s of infected farms were frequently above 20 days, and in many cases temperatures did not allow the completion of the *EIP*.

**Fig 5:**
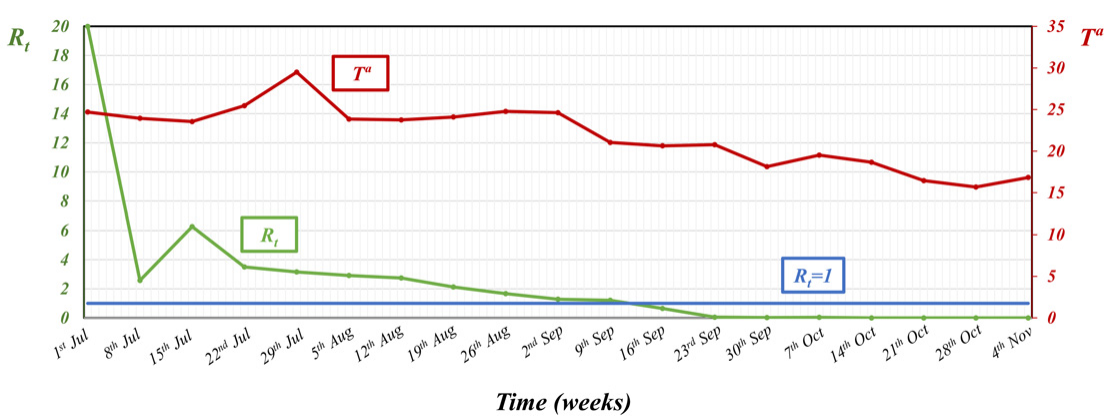
Temporal patterns of a) the weekly *R*_*t*_ values for the whole BTV-1 epidemic in Andalusia, and b) the mean weekly temperatures at the farms affected, between week 1 (1^st^ of July) and week 19 (4^th^ of November).

Fig 5 shows the relationship between the weekly *R*_*t*_ values for the whole epidemic and the mean weekly temperatures at the farms affected by the epidemic. For the first half of the epidemic (between the 1^st^ of July and the 2^nd^ of September) temperatures were more or less constant at approximately 25°C, while *R*_*t*_ values showed a decreasing trend. In contrast, for the second half of the epidemic (between the 9^th^ of September and the 4^th^ of November) decreasing mean temperatures coincided with a further decrease of *R*_*t*_ values, which fell below the threshold of 1 when temperatures dropped below 21°C. We found a positive correlation between temperature and the *R*_*t*_ values (*r* = 0.44), which was very close to significant (p = 0.06).

Correlation analysis of the different environmental predictors potentially related to *R*_*t*_ showed that the estimated median maximum abundance of *C*. *imicola* was correlated with the 95% upper limit of the credible interval of estimated maximum abundance of *C*. *imicola*, as well as with the median and 95% upper limit of the credible interval for total *Culicoides* (as *C*. *imicola* represented more than 99% of the captures in the affected areas of Andalusia). Therefore, only median estimated maximum abundance of *C*. *imicola* was retained for further analysis. There was also a positive correlation between slope and elevation (Spearman’s rank correlation = 0.99), therefore only slope was retained.

Considering the relationship between *R*_*t*_ and the predictors, support in the data for the best model was split amongst several models; with 16 models receiving approximately equal support in the data (delta AICc value of <2.0; S1 Table). Of these top 16 models, all models included the proportion of area overlapped and sheep density, 14 models included *EIP*, 8 models included red deer habitat suitability, and 7 models included slope (S3 Table). The best fitting model included these five predictors, of which proportion of area overlapped, *EIP* and sheep were strongly significant predictors of *R*_*t*_, and red deer suitability was close to significant (Table 1).

**Table 1:** Parameter estimates from the best-fitting mixed effects model used to evaluate the relative influence of different environmental variables on the estimated value of *R*_*t*_.

**Random effects:**	**Standard Deviation**			
*Focus*	*0*.*00017*			
*Week*	*4*.*38*			
*Residual*	*0*.*029*			
**Fixed effects:**	**Estimate **	**Standard Error**	***t***	***P***
*EIP*	*-0*.*0041*	*0*.*0020*	*-2*.*05*	*0*.*041*
*Slope*	*0*.*49*	*0*.*29*	*1*.*66*	*0*.*098*
*Sheep density (animals/km*^*2*^*)*	*0*.*14*	*0*.*044*	*3*.*20*	*0*.*0015*
*Overlapping (proportion)*	*-2*.*64*	*0*.*76*	*-3*.*48*	*0*.*0006*
*Red deer suitability*	*-0*.*10*	*0*.*056*	*-1*.*79*	*0*.*074*

For each day of increase in the duration of the *EIP* of the farms within a given focus, the value of *R*_*t*_ was reduced by 0.0041. For each increase of 1% in the area overlapped, the value of *R*_*t*_ was reduced by 2.64. For each unit increase in the number of sheep per km^2^, the value of *R*_*t*_ was increased by 0.14. Red deer suitability showed a weak negative correlation with *R*_*t*_ values, although it was not statistically significant.

The random effects components of the model indicated that residual spatial variability between foci not accounted for by fixed effects was much lower (SD: 0.00017; Table 1) than residual temporal variability through time (weeks) within the different foci (SD: 4.38; Table 1). Overall residual variation not attributed to spatial variation between foci or temporal variation within foci was 0.029 (Table 1).

**Fig 6:**
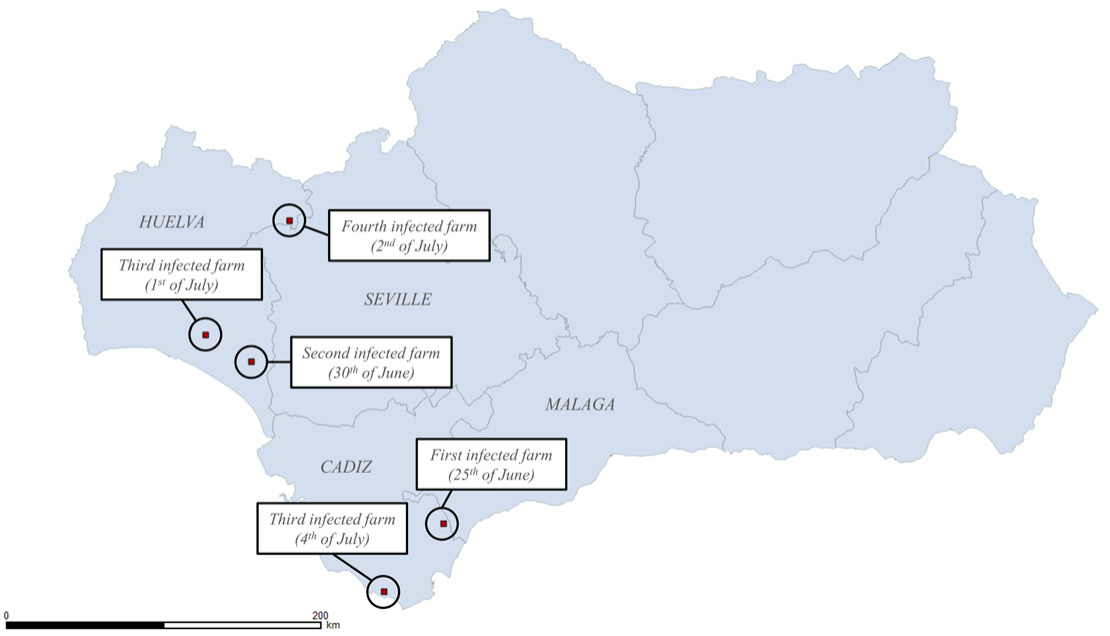
Dates of infection and location of the first 5 farms infected during the BTV-1 epidemic in Andalusia in 2007.

Based on our estimations, the infection of the first farm occurred in the South-East of the province of Cadiz on the 25^th^ of June (Fig 6). The second farm was infected 5 days later (30^th^ of June), but it was located at 128 km from the first infected farm, in the South-East of the province of Huelva. The third farm was infected on the 1^st^ of July, in the South of Huelva, 26.8 km away from the previous farm. On the 2^nd^ of July, a fourth farm was infected, also in Huelva, but in the North-East of the province, 71.3 km away from the closest infected farm at that time. The fifth farm was infected on the 4^th^ of July in Tarifa, in the southernmost area of the province of Cadiz, but 46 km away from the first infected farm. However, taking into account the temperatures on those dates on those farms, which determine the time needed for the completion of the *EIP*, it seems that none of those farms became infectious before the 5^th^ of July, and therefore were not epidemiologically linked.

**Fig 7:**
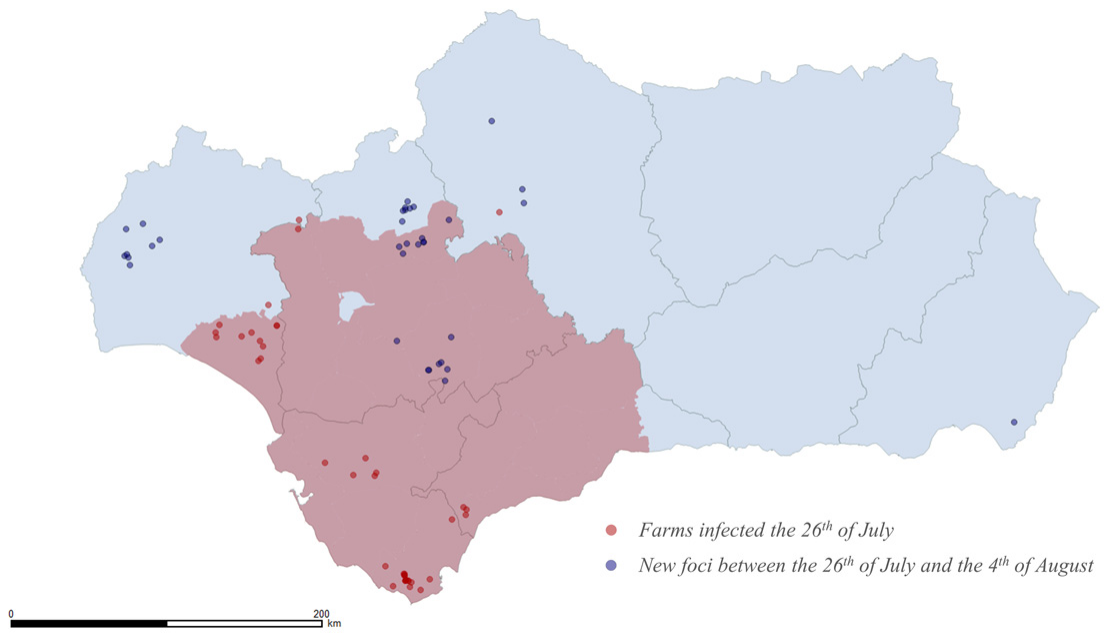
Movement restriction area established on the 26^th^ of July (red area), with the farms estimated to be infected by that date (red dots) and the new foci initiated within the 10 days that followed movement restrictions (blue dots).

The abovementioned case in Tarifa (fifth infected farm) was actually the first case of BTV-1 confirmed by the Spanish Ministry of Agriculture (on the 25^th^ of July 2007). By the 26^th^ of July movement restrictions were imposed in Cadiz, the majority of Malaga and Seville, and the eastern area of Huelva [27] (Fig 7). However, by that date, BTV-1 had spread to other areas of Andalusia, including 3 farms outside restricted areas (Fig 7).

Despite movement restrictions, 35 of the 58 foci identified throughout the whole epidemic, were initiated during the 10 days that followed the implementation of those measures (Fig 3 and Fig 7), with appearance of new foci in the western area of Huelva and the previously unaffected provinces of Cordoba and Almeria period. Although the majority of farms infected throughout the epidemic were located in Western Andalusia, there were foci in the 8 provinces of Andalusia.

## Discussion

This study represents the first time that spatial and temporal variability in the case-reproduction ratio has been estimated for a midge-borne disease and related to the variability in key host, climate and landscape factors across the invaded region. Here, we discuss the implications of these findings for management of future disease incursions, both in Andalusia, a critical point of entry for bluetongue virus strains into Europe, and further afield.

The evaluation of the evolution of the monthly case-reproduction ratio of the BTV-1 epidemic for the whole of Andalusia evidenced that *R*_*t*_ declined progressively: from 4.6 in July, to 2.2 in August, such that by September *R*_*t*_ fell below the key threshold of 1 (value of 0.8), and by October the value was close to 0. De Koeijer and collaborators [28] estimated the temporal pattern of the basic reproduction number between herds for the BTV-8 epidemic in Northern Europe, and they obtained a value of around 4 for the second half of the summer, which progressively declined, falling below 1 by autumn. Values in Andalusia were therefore very similar to those obtained in Northern Europe despite differences in the epidemiology of BTV between the two areas, including the fact that the main vector in Andalusia is *Culicoides imicola*, while in Northern Europe is *Culicoides obsoletus*.

In contrast to the gradual decrease of *R*_*t*_, the number of infected farms progressively increased, from only 113 farms infected in July to a maximum of 2089 in September, and remained high in October (1192) despite the low value of *R*_*t*_ that month. This apparent discrepancy between *R*_*t*_ and the number of infected farms may be explained by the fact that, as the epidemic advances, more and more farms become infectious, which results in an increase of the number of farms infected, but also an increase of the number of potential parents for those farms, and therefore a decrease of the values of *R*_*t*_. There were no significant differences between the *R*_*t*_ values obtained by the three different algorithms used for the determination of parent farms on the basis of the distance. Regardless of the algorithm, *R*_*t*_ values for a given focus area and period are averaged across that area and period, and therefore whether the parenthood is assigned to a given farm or its neighbour does not have a great effect on the estimate of *R*_*t*_ for that area and period. Therefore, we can be fairly confident in the reliability of the case-reproduction ratio estimated for the different areas and periods. However, a relevant difference is that when algorithm 1 (closest infectious farm) was applied, the number of potential parent farms was much lower as compared to when algorithm 2 (equal probability within a distance) was applied, as in that case, the probability of infection was shared among all the infectious farms within 5 kilometers. With algorithm 1, those particular farms, especially the farms that initiate the new foci, tended to be responsible for the infection of a great number of farms (i.e. they act as super-spreaders). In fact, with algorithm 1, the 20% most infective farms infected 87% of the total farms affected throughout the whole epidemic, close to the 20/80 rule (i.e. 20% of the population contributes to 80% of the net transmission) which applies in many disease systems [6]. That may be relevant in relation to the control of the disease, if we were able to achieve early identification of those farms, control measures would be much more effective [6]. In fact, with algorithm 1, 67% of the farms did not infect any farm, a value which is also very similar to the 70% of “end-point” farms obtained by Sedda and collaborators [18] for the BTV-8 epidemic in Northern Europe. When algorithm 2 was applied, the 20% most infective farms infected only 56% of the total farms affected throughout the whole epidemic, and only 10% of the farms were considered “end-point” farms, i.e. they have a zero probability of infecting other farms. However, for the majority of the remaining 90% farms, the probabilities of infecting other farms were very low. Finally, the values obtained with algorithm 3 (proportional to distance) were similar to those obtained with algorithm 1, as the 20% most infective farms were responsible for the infection of 88% of the total farms affected, and 62% of the farms did not contribute to any further infections.

When we used a smaller time-step (i.e. one week) to evaluate the temporal pattern of the *R*_*t*_ values for the whole BTV-1 epidemic in Andalusia, we observed an increase in the variability of the case-reproduction ratio, which went from 20 in the first week, to 2.6 in the second and then to 6.3 in the third week. That heterogeneity can be more easily understood if we look at the epidemic not as a whole, but as a set of different foci, and realise that those global *R*_*t*_ values are simply the result of the complex combination of both of local BTV transmission within established foci and BTV spread to new (distant) areas, i.e. new foci, where BTV may spread more or less efficiently depending on different factors. The mechanism by which BTV-1 jumped to those new distant areas in Andalusia to establish new foci, which implied travelling distances over 52.1 kilometers, remains uncertain. In the 2006 BTV-8 epidemic in northwest Europe, 2% of infections seemed to have occurred at distances over 31 kilometers, probably related to wind dispersal of *Culicoides*, although other mechanisms such as human transport of midges or virus, or movement of domestic or wild animals, could not be ruled out [18]. Unless we are able to clarify those mechanisms of long-distance dispersal, the prediction of disease spread and the establishment of effective control measures will be hampered.

The heterogeneity of BTV spread through time and space means that control measures applied on the basis of global parameters (e.g. vaccination of a proportion of the population calculated from the *R*_*t*_ value for the whole epidemic) would probably result in measures more exhaustive than needed in some areas (with the subsequent waste of resources), while in other areas the same measures will be insufficient to control the disease. This highlights the need for more detailed understanding of spatio-temporal variation in transmission as epidemics progress.

The delay observed in many foci between the initial infection and the secondary cases arising as a result of local spread may have been due to a lack of suitable conditions for disease spread in that period or a failure of detection of those secondary cases. In addition, if the new focus resulted from the introduction of a recently infected host (rather than infected vectors), before local transmission could occur, the infected host needs to reach the viraemic stage and then the local vectors need to complete the *EIP* before onwards transmission can occur. The fact that the duration of the *EIP*s increased progressively throughout the epidemic, seems to indicate that the decrease in temperature may have played a more relevant role in stopping BTV-1 spread than vaccination (which mainly was applied from November onwards). However, vaccination was probably effective at preventing the persistence of the disease in the following year: only 10 cases of BTV-1 were reported in Andalusia in 2008, and they occurred by mid-October, when BTV-1 was widespread in other regions of Spain.

Within the range of values at which BTV-1 transmission occurred in Andalusia (i.e., between 20.6°C and 29.5°C) there was a general positive correlation between temperature and *R*_*t*_ values (i.e., the higher the temperature, the higher the *R*_*t*_ values). However, our results indicated that the relationship was not linear, but had a more complicated pattern. This pattern is probably the result of the complex relationship between temperature and different parameters affecting BTV transmission. While higher temperatures increase the virogenesis rate (i.e., reduce the *EIP*) and the biting rate, and therefore favor BTV transmission, they also increase the adult vector mortality rate (i.e., reduce the *Culicoides* lifespan), which hampers the transmission of BTV (as reviewed in Gubbins and collaborators [29]). As well as influencing parameters that determine BTV transmission, temperature is also one of the main determinants of *Culicoides* abundance, though the effect of temperature may interact with moisture availability [30]. *R*_*t*_ values for BTV-1 in Andalusia fell below the threshold of 1 when temperatures dropped below 21°C. In contrast, in the BTV-8 epidemic in Northern Europe, the reproduction ratio between herds was estimated to drop below 1 only when temperatures fell below 15°C [31]. This divergence may be explained by differences in the adaptation to temperature of the main vectors of the BTV-1 epidemic in Andalusia (*Culicoides imicola*) and the BTV-8 epidemic in Northern Europe (*Culicoides obsoletus*). The distribution and abundance of *C*. *imicola* seems to be constrained by their relatively poor tolerance of lower temperatures [32]. On the other hand, *C*. *imicola* Kieffer infected with BTV-1 showed no apparent transmission potential at 15°C [33].

The results of the mixed effects model indicated that variability through time (weeks) within the different foci was significantly higher than among different spatial foci. This is likely to be related to the importance of temperature on BTV transmission, which is consistent with the fact that the duration of *EIP* was found to be significantly associated with the rate of spread. The significance of overlapping foci indicates the relevance of this phenomenon in BTV transmission in Andalusia. Overlapping of disease foci may result in the underestimation of the *R*_*t*_ value for a given focus because some of the farms within its area may be infected by farms of another (overlapping) focus, which needs to be taken into account when interpreting the *R*_*t*_ values. Finally, the significant increase of *R*_*t*_ with sheep density is an indication that this species was the key host in the BTV-1 epidemic in Andalusia. The negative correlation between red deer suitability and *R*_*t*_ values is in contradiction with previous studies [10,34]. However, this result should be interpreted with care as the correlation was weak and not statistically significant, and negativity may have been associated to the fact that many of the areas with the highest suitability are located in areas that were invaded later in the epidemic, when the conditions for BTV transmission were less favourable.

Based on the estimated dates of infection, it seems that the first five farms affected in the epidemic were infected between the 25^th^ of June and the 4^th^ of July. However, taking into account the temperatures on those dates on those farms, which determine the time needed for the completion of the *EIP*, it seems that none of those farms became infectious before the 5^th^ of July. This almost simultaneous infection and the fact that these farms were located far away from each other, suggests that the BTV-1 epidemic in Andalusia in 2007 may have not been the result of a single introduction, but several almost simultaneous introductions at distant locations. In the summer of 2007 BTV-1 was circulating in Morocco, and *Culicoides* carried on the wind were considered as the most likely explanation for the introduction of BTV-1 into Spain, as neither bovines nor small ruminants were reported to have been imported from Morocco [10]. Therefore, BTV-1 infected *Culicoides* from Morocco may have been transported to these distant locations, where local transmission resulted in the initiation of different disease foci. If BTV-1 (and previously other serotypes) were believed to be introduced into Andalusia through wind transportation of infected vectors from the north of Africa, it would be reasonable to think that that phenomenon occurred not only at a single point at the start of the epidemic, but at several times and different locations throughout the epidemic. Whether wind transportation of infected vectors from the north of Africa actually occurs, and if it does, its frequency deserves further investigation, because that would have a great influence on how BTV spreads and therefore on the effectiveness of control measures.

Even though movement restrictions were imposed the day after the confirmation of the first outbreak in an extensive area around the first confirmed focus, by that time, BTV-1 had spread to other areas of Andalusia, including some regions outside restricted areas. Therefore, it seems clear that the movement restrictions imposed in Andalusia were not able to stop the spread of the disease. For example, 35 out of the 58 foci of the epidemic were reported during the 10 days that followed those restrictions. Long distance transmission, which results in new foci, may be the result of the introduction of infected vectors into the new area (e.g. transported on the wind) or of infected hosts, either domestic or wild. Further studies would be needed to clarify the relative importance of the different mechanisms of long distance spread in Andalusia, which would allow the implementation of more effective measures for the control of the disease. Failure of movement restrictions to stop BTV spread is in accordance with previous BTV epidemics, such as the BTV-8 epidemic in Northern Europe [35]. However, it is reasonable to think that if movement of animals had not been forbidden, the spread of disease would have been greater.

Some of the limitations of the study are related to the fact that the detection relied on clinical suspicion. Some lack of accuracy in the dates of infection of the farms should be expected and that may influence the results. Besides, some underreporting would be expected, although we assumed that veterinary inspection was homogeneous over the territory and therefore the different areas would be equally affected by underreporting. This assumption is supported by previous studies, for example Méroc and collaborators [36], concluded that the surveillance system based on clinical detection underestimated the real impact of the BTV epidemic, but described accurately the spatial distribution of the virus. Another problem was that as neither clinical signs nor mortality were observed in cattle, cattle herds had to be excluded from the analysis. This limitation probably did not have a great influence on the spatial distribution of the epidemic, as sheep are widely distributed and BTV-1 spread to all the provinces in Andalusia. In contrast, underreporting in cattle may have biased the estimation of the intensity of transmission in some areas, although the analysis showed that the density of cattle did not have a significant effect on the level of spread, in contrast to the density of sheep. Moreover, the cattle population represents only a 13.6% (0.6 out of 4.4 million) of the total ruminant population in Andalusia, and of the 4421 affected farms, 722 were mixed farms (sheep, goat and/or cattle in the same farm), which were included in the analysis.

In order to assess the effect of vectors we used estimates of the median annual maximum abundance derived from an analysis of national surveillance weekly trapping data. However, using a static estimate of vector abundance ignores how seasonal variation in *Culicoides* numbers throughout the year may have affected transmission. In fact a drop off in vector numbers in autumn may be coincident with the lengthening of the extrinsic incubation period found to significantly reduce *R*_*t*_ in this period. Moreover, the large differences in *Culicoides* catches between neighbouring farms [37] indicate the limitations of extrapolating the results of entomological surveillance systems to describe the abundance of *Culicoides* in nearby areas. This may be why we detected no significant effect of vector abundance on the spread of BTV in the epidemic. Other estimates of *R*_*0*_ value for BTV [29,38,39,40] have largely considered animals as units, rather than farms (i.e. average number of secondary cases arising from the introduction of a single infected individual into a totally susceptible population) and therefore assume that animals are homogeneously distributed, rather than clustered in farms. The models by Gubbins and collaborators [29], Hartemink and collaborators [38] and Guis and collaborators [39] are theoretical models using the density of hosts and vectors, plus several assumptions about transmission parameters. Since European *Culicoides* vectors are diverse, taxonomically cryptic and difficult to colonise the range of variability of many of these transmission parameters is poorly described [41]. By contrast Santman-Berends and collaborators [40], estimated *R*_*0*_ using serological field data from the BTV-8 epidemic in the Netherlands, but these data are expensive to collect and highly dependent on how well the sites selected for surveillance represent relevant environmental drivers. While Hartemink and collaborators [38] considered variability of *R*_*0*_ across space and seasons, the final predictions are highly dependent on input geographical data for densities of animal hosts and vectors with their associated uncertainties. We argue that estimating the between-herd reproduction ratio for bluetongue directly from empirical epidemic data provides the best opportunity for understanding spatial and temporally variability in transmission and for linking this variability to environmental conditions. This is because relatively few simplifying assumptions and entomological parameters are required and a wide spectrum of environmental conditions that may impact BTV is potentially encompassed within farms across the whole epidemic region.

## Conclusions

BTV-1 epidemic in Andalusia resulted from a combination of local transmission and long distance jumps with the establishment of new foci that produced large variability in transmission, not only through time, but also among the different geographical areas. This heterogeneity means that area-wide control measures are unlikely to be effective. Transmission was favoured by the increase in the density of sheep and by the reduction of the *EIP* (which is dependent on the temperature). There seemed to be a general positive correlation between temperature and *R*_*t*_ values, although the relationship was not linear, probably as a result of the complex relationship between temperature and the different parameters affecting BTV transmission. *R*_*t*_ values for BTV-1 in Andalusia fell below the threshold of 1 when temperatures dropped below 21°C, while for BTV-8 epidemic in Northern Europe, that occurred at 15°C. This divergence may be explained by differences in the adaptation to temperature of the main vectors of the BTV-1 epidemic in Andalusia (*Culicoides imicola*) and the BTV-8 epidemic in Northern Europe (*Culicoides obsoletus*).

Control of BTV-1 in Andalusia was further complicated by the simultaneous establishment of the disease at several distant foci, which may have been caused by the repeated introductions of infected vectors at distant locations. Therefore, whenever orbiviral diseases are circulating in the North of Africa, surveillance in Andalusia should be intensified (and extended beyond the closer areas), as the south of Spain is one of the main entry point for these diseases into Europe.

## Supporting Information

S1 Fig Steps in the calculation of the case reproduction ratio (*R*_*t*_): 1- Determination of the date of infection and the date of onset of infectiousness of the affected farms, 2- Development of the epidemic trees, 3- Calculation of the number of farms infected by each of the farms affected throughout the epidemic, and 4- Calculation of the case-reproduction ratio (*R*_*t*_) for a given focus (x) and time period (t).
(TIF)

S2 Fig Detailed description of the calculations of the date of infection and the date of onset of infectiousness of the affected farms. (TIF)

S1 Table Summary of all predictors used in the mixed-effects model. Information includes the parameters used, their units and the sources of data.
(DOCX)

S2 Table Assignment of CORINE landcover classes to the three categories describing the cost of movement by vectors over different landscapes. (DOCX)

S3 Table Parameter estimates and model fitting statistics (AICc and model weights) for all models within 3 AICc units of the best-fitting model. (DOCX)
