# Supplementary material for: Understanding Spatio-Temporal Variability in the Reproduction Ratio of the Bluetongue (BTV-1) Epidemic in Southern Spain (Andalusia) in 2007 Using Epidemic Trees
Source: PLoS One. 2016 Mar 10;11(3):e0151151. doi: 10.1371/journal.pone.0151151 (PMC4786328; doi:10.1371/journal.pone.0151151)
Supplement: S1 Table — Information includes the parameters used, their units and the sources of data. (DOCX) [file pone.0151151.s003.docx]

S1 Table: Summary of all predictors used in the mixed-effects model. Information includes the parameters used, their units and the sources of data.* *Robert J. Hijmans (2015). raster: Geographic Data Analysis and Modeling. R package version 2.5-2.*

| **PARAMETER** | **UNITS** | **SOURCE** |
| --- | --- | --- |
| **Variables related to the host species** |  |  |
| Density of cattle | Cattle herds per km^2^ | Department of Agriculture of the Autonomous Government of Andalusia |
| Density of sheep | Sheep flocks per km^2^ | Department of Agriculture of the Autonomous Government of Andalusia |
| Density of goats | Goat herds per km^2^ | Department of Agriculture of the Autonomous Government of Andalusia |
| Red deer habitat suitability | predicted habitat suitability (probability) | Wint et al., 2014 |
| Roe deer habitat suitability | predicted habitat suitability (probability) | Alexander et al., 2015 |
| **Variables related to the vector species** |  |  |
| Density of *C. imicola* (Median) | Predicted maximum trap catch (*C. imicola*) per km^2^ | Searle et al., 2015 |
| Density of *C. imicola* (Upper limit of the 95% credible interval) | Predicted maximum trap catch (*C. imicola*) per km^2^ | Searle et al., 2015 |
| Density of *C. obsoletus* (Median) | Predicted maximum trap catch (*C. obsoletus*) per km^2^ | Searle et al., 2015 |
| Density of *C. iobsoletus* (Upper limit of the 95% credible interval) | Predicted maximum trap catch (*C. obsoletus*) per km^2^ | Searle et al., 2015 |
| Density of *C. pulicaris* (Median) | Predicted maximum trap catch (*C. pulicaris*) per km^2^ | Searle et al., 2015 |
| Density of *C. pulicaris* (Upper limit of the 95% credible interval) | Predicted maximum trap catch (*C. pulicaris*) per km^2^ | Searle et al., 2015 |
| Density of competent *Culicoides* species (Median) | Predicted maximum trap catch (*C. imicola + C. obsoletus + C. pulicaris*) per km^2^ | Searle et al., 2015 |
| Density of competent *Culicoides* species (Upper limit of the 95% credible interval) | Predicted maximum trap catch (*C. imicola + C. obsoletus + C. pulicaris*) per km^2^ | Searle et al., 2015 |
| **Other variables** |  |  |
| Median *EIP* | Days | Temperatures & Napp et al., 2011 |
| Time since the start of each focus | Days | Model |
| Proportion of the area of the focus overlapped by other foci (for a given focus and time step) | NA | Model |
| Elevation | Metres | Jarvis et al., 2008 |
| Slope | Angle (degrees) | Hijmans (2015)* |
| Terrain ruggedness index - elevation | Mean of the absolute differences between the value of a cell and the value of its eight surrounding cells | Hijmans (2015)* |
| Terrain ruggedness index - slope | Mean of the absolute differences between the value of a cell and the value of its eight surrounding cells | Hijmans (2015)* |
