# Supplementary material for: Understanding Spatio-Temporal Variability in the Reproduction Ratio of the Bluetongue (BTV-1) Epidemic in Southern Spain (Andalusia) in 2007 Using Epidemic Trees
Source: PLoS One. 2016 Mar 10;11(3):e0151151. doi: 10.1371/journal.pone.0151151 (PMC4786328; doi:10.1371/journal.pone.0151151)
Supplement: S2 Table — (DOCX) [file pone.0151151.s004.docx]

S2 Table. Assignment of CORINE landcover classes to the three categories describing the cost of movement by vectors over different landscapes.

| CORINE LANDCOVER CLASS CODE | COST OF VECTOR MOVEMENT | Level 3 | Level 2 | Level 1 | % of Andalusian farms found in landcover class |
| --- | --- | --- | --- | --- | --- |
| 211 | 0 | Non-irrigated arable land | Arable land | Agricultural areas | 17.19 |
| 212 | 0 | Permanently irrigated land | Arable land | Agricultural areas | 5.36 |
| 223 | 0 | Olive groves | Permanent crops | Agricultural areas | 8.76 |
| 242 | 0 | Complex cultivation patterns | Heterogeneous agricultural areas | Agricultural areas | 13.03 |
| 243 | 0 | Land principally occupied by agriculture, with significant areas of natural vegetation | Heterogeneous agricultural areas | Agricultural areas | 5.14 |
| 244 | 0 | Agro-forestry areas | Heterogeneous agricultural areas | Agricultural areas | 17.13 |
| 321 | 0 | Natural grasslands | Scrub and/or herbaceous vegetation associations | Forest and semi natural areas | 5.42 |
| 323 | 0 | Sclerophyllous vegetation | Scrub and/or herbaceous vegetation associations | Forest and semi natural areas | 5.68 |
| 324 | 0 | Transitional woodland-shrub | Scrub and/or herbaceous vegetation associations | Forest and semi natural areas | 5.88 |
| 333 | 0 | Sparsely vegetated areas | Open spaces with little or no vegetation | Forest and semi natural areas | 1.66 |
| 221 | 1 | Vineyards | Permanent crops | Agricultural areas | 0.12 |
| 222 | 1 | Fruit trees and berry plantations | Permanent crops | Agricultural areas | 2.22 |
| 231 | 1 | Pastures | Pastures | Agricultural areas | 0.02 |
| 241 | 1 | Annual crops associated with permanent crops | Heterogeneous agricultural areas | Agricultural areas | 0.04 |
| 311 | 1 | Broad-leaved forest | Forests | Forest and semi natural areas | 5.76 |
| 312 | 1 | Coniferous forest | Forests | Forest and semi natural areas | 1.27 |
| 313 | 1 | Mixed forest | Forests | Forest and semi natural areas | 0.41 |
| 111 | 2 | Continuous urban fabric | Urban fabric | Artificial surfaces | 2.26 |
| 112 | 2 | Discontinuous urban fabric | Urban fabric | Artificial surfaces | 0.89 |
| 121 | 2 | Industrial or commercial units | Industrial, commercial and transport units | Artificial surfaces | 0.22 |
| 122 | 2 | Road and rail networks and associated land | Industrial, commercial and transport units | Artificial surfaces | 0.01 |
| 123 | 2 | Port areas | Industrial, commercial and transport units | Artificial surfaces | 0.00 |
| 124 | 2 | Airports | Industrial, commercial and transport units | Artificial surfaces | 0.03 |
| 131 | 2 | Mineral extraction sites | Mine, dump and construction sites | Artificial surfaces | 0.17 |
| 132 | 2 | Dump sites | Mine, dump and construction sites | Artificial surfaces | 0.01 |
| 133 | 2 | Construction sites | Mine, dump and construction sites | Artificial surfaces | 0.25 |
| 141 | 2 | Green urban areas | Artificial, non-agricultural vegetated areas | Artificial surfaces | 0.00 |
| 142 | 2 | Sport and leisure facilities | Artificial, non-agricultural vegetated areas | Artificial surfaces | 0.04 |
| 213 | 2 | Rice fields | Arable land | Agricultural areas | 0.05 |
| 331 | 2 | Beaches, dunes, sands | Open spaces with little or no vegetation | Forest and semi natural areas | 0.04 |
| 332 | 2 | Bare rocks | Open spaces with little or no vegetation | Forest and semi natural areas | 0.05 |
| 334 | 2 | Burnt areas | Open spaces with little or no vegetation | Forest and semi natural areas | 0.04 |
| 411 | 2 | Inland marshes | Inland wetlands | Wetlands | 0.50 |
| 421 | 2 | Salt marshes | Maritime wetlands | Wetlands | 0.05 |
| 422 | 2 | Salines | Maritime wetlands | Wetlands | 0.04 |
| 423 | 2 | Intertidal flats | Maritime wetlands | Wetlands | 0.00 |
| 511 | 2 | Water courses | Inland waters | Water bodies | 0.06 |
| 512 | 2 | Water bodies | Inland waters | Water bodies | 0.19 |
| 521 | 2 | Coastal lagoons | Marine waters | Water bodies | 0.00 |
| 522 | 2 | Estuaries | Marine waters | Water bodies | 0.00 |
| 523 | 2 | Sea and ocean | Marine waters | Water bodies | 0.01 |
