# Supplementary material for: Understanding Spatio-Temporal Variability in the Reproduction Ratio of the Bluetongue (BTV-1) Epidemic in Southern Spain (Andalusia) in 2007 Using Epidemic Trees
Source: PLoS One. 2016 Mar 10;11(3):e0151151. doi: 10.1371/journal.pone.0151151 (PMC4786328; doi:10.1371/journal.pone.0151151)
Supplement: S3 Table — (DOCX) [file pone.0151151.s005.docx]

S3 Table: Parameter estimates and model fitting statistics (AICc and model weights) for all models within 3 AICc units of the best-fitting model.

| **Intercept** | ***C. imicola (median)*** | **Overlapping (proportion)** | ***EIP*** | **Goat density** | **Land-cover** | **Red deer suitability** | **Roe deer suitability** | **Sheep density** | **Slope** | **df** | **logLik** | **AICc** | **delta** | **weight** |
| --- | --- | --- | --- | --- | --- | --- | --- | --- | --- | --- | --- | --- | --- | --- |
| -0.8473 |  | -2.65 | -0.0041 |  |  | -0.0990 |  | 0.1406 | 0.4925 | 9 | -1109.64 | 2237.76 | 0.00 | 0.0707 |
| -0.4113 |  | -2.57 | -0.0035 |  |  |  |  | 0.1044 |  | 7 | -1111.90 | 2238.09 | 0.33 | 0.0598 |
| -0.3490 |  | -2.63 | -0.0036 |  |  | -0.0681 |  | 0.1374 |  | 8 | -1111.05 | 2238.49 | 0.73 | 0.0491 |
| -0.9935 |  | -2.92 | -0.0035 |  |  |  | -0.0560 | 0.1436 |  | 8 | -1111.07 | 2238.52 | 0.77 | 0.0482 |
| -15.330 |  | -2.98 | -0.0039 |  |  |  | -0.0673 | 0.1420 | 0.3954 | 9 | -1110.08 | 2238.64 | 0.88 | 0.0455 |
| -0.7517 |  | -2.56 | -0.0038 |  |  |  |  | 0.0967 | 0.3183 | 8 | -1111.24 | 2238.86 | 1.10 | 0.0408 |
| -13.590 |  | -2.92 | -0.0041 |  |  | -0.0796 | -0.0457 | 0.1627 | 0.5106 | 10 | -1109.16 | 2238.92 | 1.16 | 0.0396 |
| -17.043 |  | -2.78 | -0.0038 |  | 0.0779 | -0.0797 |  | 0.1353 |  | 9 | -1110.26 | 2239.01 | 1.25 | 0.0378 |
| -14.921 |  | -2.67 | -0.0037 |  | 0.0617 |  |  | 0.0983 |  | 8 | -1111.39 | 2239.16 | 1.41 | 0.0350 |
| -0.3557 |  | -2.40 |  |  |  |  |  | 0.0985 |  | 6 | -1113.51 | 2239.24 | 1.48 | 0.0337 |
| -14.659 |  | -2.72 | -0.0041 |  | 0.0404 | -0.0999 |  | 0.1390 | 0.4100 | 10 | -1109.47 | 2239.52 | 1.76 | 0.0293 |
| -14.067 |  | -2.54 | -0.0037 | 0.0508 |  |  |  | 0.1206 |  | 8 | -1111.58 | 2239.54 | 1.78 | 0.0290 |
| -16.208 |  | -2.61 | -0.0038 | 0.0652 |  | -0.0764 |  | 0.1623 |  | 9 | -1110.54 | 2239.55 | 1.80 | 0.0288 |
| -11.297 | 0.000015 | -2.65 | -0.0040 |  |  | -0.1012 |  | 0.1456 | 0.5374 | 10 | -1109.51 | 2239.62 | 1.86 | 0.0279 |
| -0.9340 |  | -2.76 |  |  |  |  | -0.0557 | 0.1375 |  | 7 | -1112.70 | 2239.69 | 1.94 | 0.0268 |
| -12.682 |  | -2.64 | -0.0041 | 0.0240 |  | -0.0992 |  | 0.1494 | 0.4463 | 10 | -1109.58 | 2239.75 | 1.99 | 0.0261 |
| -0.2947 |  | -2.46 |  |  |  | -0.0655 |  | 0.1302 |  | 7 | -1112.73 | 2239.76 | 2.00 | 0.0260 |
| -0.7863 |  | -2.87 | -0.0036 |  |  | -0.0498 | -0.0405 | 0.1569 |  | 9 | -1110.68 | 2239.84 | 2.08 | 0.0249 |
| -0.6995 |  | -2.46 |  |  |  | -0.0907 |  | 0.1320 | 0.4058 | 8 | -1111.76 | 2239.91 | 2.15 | 0.0241 |
| -29.799 |  | -2.76 | -0.0040 | 0.0654 | 0.0780 | -0.0881 |  | 0.1603 |  | 10 | -1109.74 | 2240.08 | 2.32 | 0.0221 |
| -17.017 |  | -2.95 | -0.0036 |  | 0.0451 |  | -0.0482 | 0.1337 |  | 9 | -1110.81 | 2240.11 | 2.35 | 0.0218 |
| -18.628 |  | -2.89 | -0.0037 | 0.0454 |  |  | -0.0539 | 0.1567 |  | 9 | -1110.81 | 2240.11 | 2.35 | 0.0218 |
| -20.907 | 0.000024 | -3.04 | -0.0039 |  |  |  | -0.0764 | 0.1547 | 0.4721 | 10 | -1109.78 | 2240.15 | 2.39 | 0.0214 |
| -0.4148 | 0.000000 | -2.57 | -0.0035 |  |  |  |  | 0.1044 |  | 8 | -1111.90 | 2240.18 | 2.42 | 0.0211 |
| -13.701 |  | -2.79 |  |  |  |  | -0.0648 | 0.1357 | 0.3233 | 8 | -1112.03 | 2240.45 | 2.69 | 0.0184 |
| -19.077 | 0.000023 | -2.97 | -0.0040 |  |  | -0.0791 | -0.0548 | 0.1750 | 0.5853 | 11 | -1108.87 | 2240.46 | 2.70 | 0.0183 |
| -0.6200 |  | -2.38 |  |  |  |  |  | 0.0922 | 0.2504 | 7 | -1113.10 | 2240.49 | 2.73 | 0.0180 |
| -12.953 |  | -2.49 |  |  | 0.0537 |  |  | 0.0930 |  | 7 | -1113.12 | 2240.55 | 2.79 | 0.0175 |
| -11.370 | 0.000007 | -2.94 | -0.0035 |  |  |  | -0.0582 | 0.1477 |  | 9 | -1111.04 | 2240.55 | 2.80 | 0.0175 |
| -0.3379 | -0.000001 | -2.63 | -0.0036 |  |  | -0.0681 |  | 0.1371 |  | 9 | -1111.05 | 2240.58 | 2.83 | 0.0172 |
| -14.907 |  | -2.58 |  |  | 0.0689 | -0.0756 |  | 0.1280 |  | 8 | -1112.12 | 2240.62 | 2.86 | 0.0169 |
| -13.205 |  | -2.62 | -0.0038 |  | 0.0372 |  |  | 0.0949 | 0.2410 | 9 | -1111.09 | 2240.66 | 2.91 | 0.0165 |
| -24.343 |  | -2.65 | -0.0038 | 0.0491 | 0.0604 |  |  | 0.1141 |  | 9 | -1111.09 | 2240.66 | 2.91 | 0.0165 |
| -16.116 |  | -2.98 | -0.0039 | 0.0048 |  |  | -0.0668 | 0.1434 | 0.3855 | 10 | -1110.08 | 2240.74 | 2.99 | 0.0159 |
| -15.015 |  | -2.98 | -0.0039 |  | -0.0025 |  | -0.0679 | 0.1425 | 0.4013 | 10 | -1110.08 | 2240.75 | 2.99 | 0.0159 |
